# Science-based health innovation in sub-Saharan Africa

**DOI:** 10.1186/1472-698X-10-S1-S1

**Published:** 2010-12-13

**Authors:** Sara Al-Bader, Hassan Masum, Ken Simiyu, Abdallah S Daar, Peter A Singer

**Affiliations:** 1 McLaughlin-Rotman Centre for Global Health, at the University Health Network and University of Toronto, MaRS Centre, South Tower, Suite 406, 101 College Street, Toronto, Ontario, M5G 1L7, Canada

## Abstract

In recent years emerging markets such as India, China, and Brazil have developed appropriate business models and lower-cost technological innovations to address health challenges locally and internationally. But it is not well understood what capabilities African countries, with their high disease burden, have in science-based health innovation.

This gap in knowledge is addressed by this series in *BMC International Health and Human Rights*. The series presents the results of extensive on-the-ground research in the form of four country case studies of health and biotechnology innovation, six studies of institutions within Africa involved in health product development, and one study of health venture funds in Africa. To the best of our knowledge it is the first extensive collection of empirical work on African science-based health innovation.

The four country cases are Ghana, Rwanda, Tanzania and Uganda. The six case studies of institutions are A to Z Textiles (Tanzania), Acorn Technologies (South Africa), Bioventures venture capital fund (South Africa), the Malagasy Institute of Applied Research (IMRA; Madagascar), the Kenyan Medical Research Institute (KEMRI; Kenya), and Niprisan’s development by Nigeria’s National Institute for Pharmaceutical Research and Development and Xechem (Nigeria).

All of the examples highlight pioneering attempts to build technological capacity, create economic opportunities, and retain talent on a continent significantly affected by brain drain. They point to the practical challenges for innovators on the ground, and suggest potentially helpful policies, funding streams, and other support systems.

For African nations, health innovation represents an opportunity to increase domestic capacity to solve health challenges; for international funders, it is an opportunity to move beyond foreign aid and dependency. The shared goal is creating self-sustaining innovation that has both health and development impacts. While this is a long-term strategy, this series shows the potential of African-led innovation, and indicates how it might balance realism against opportunity. There is ample scope to learn lessons more systematically from cases like those we discuss; to link entrepreneurs, scientists, funders, and policy-makers into a network to share opportunities and challenges; and ultimately to better support and stimulate African-led health innovation.

## Introduction

Innovations in health technologies, including advancements in biotechnology, have led to vast health improvements in the developed world [1]. The development and commercialization of new drugs, vaccines, diagnostics, and other health technologies for the wealthier parts of the world command sizeable investment every year [2-4].

In contrast, innovation in life sciences for diseases affecting people living in the developing world has historically been low [5]. Despite the vast health and economic burden associated with disease in developing countries, particularly in sub-Saharan Africa, developed world companies have not generally ventured to address them. Historically, demand and distribution mechanisms were poorly understood, potential profits perceived to be low, and overall risk considered to outweigh reward [6].

Over the last decade, a number of initiatives have emerged to address these issues including Advance Market Commitments, product development partnerships, patent pools, and prizes to reward innovation. The number of drugs in development for neglected diseases has increased [7], and funding for research and development (R&D) for neglected diseases has increased significantly. In 2008, almost US$3.1 billion was invested in this area, with HIV/AIDS, malaria and tuberculosis initiatives comprising close to three-quarters of this investment [8].

While these new research and funding initiatives are welcome, they are mainly concerned with creating incentives for innovation within developed country institutions. Questions remain about ongoing costs and sustainability of neglected disease R&D [9], and the capacity for developing countries, particularly in Africa, to absorb new technologies within local contexts.

In recent years we have seen how emerging markets such as India, China, and Brazil are developing appropriate business models and lower-cost technological innovations to address health challenges locally and internationally [10-13]. Indeed, recent reports indicate that for some diseases, traditional donor funding is increasingly being supplemented by investment from these emerging economies [8].

A missing piece of the global health R&D puzzle is understanding what capabilities African countries themselves have in science-based health innovation – in internalizing foreign health innovations, and especially in developing their own home-grown ideas and translating these into products and services. The contribution of African countries to health innovation for their own needs is not well-documented or understood. Nor is involvement in local health product development purely a health issue: as African countries attempt to diversify their economies, it will be a high priority to build science, technology and innovation capacity and stimulate an entrepreneurial culture responsive to local needs [14,15]. Without indigenous capacity in innovation—be it R&D infrastructure, trained personnel, funding for the development and translation of new ideas, or the presence of firms that can participate in product development and delivery—it is hard to imagine that foreign technologies, however much needed, can be sustainably or affordably absorbed or utilized, or that indigenous technologies can be fostered for local use.

A series of efforts have been made over the last few years to build commitment at the policy level and mobilize resources towards home-grown African health innovation. These include the New Economic Partnership for African Development (NEPAD) Consolidated S&T Action Plan [16], which outlines key technology platforms for the continent, and the NEPAD/African Union report on Science, Technology and Innovation for Public Health in Africa, which comprehensively makes the case for health innovation systems and highlights the necessary changes in policy thinking which will be needed to tie health goals to economic ones [17]. The High-Level African Panel on Modern Biotechnology report, *Freedom to Innovate *[18], which reported to the African Union, also addressed health biotechnology and made recommendations for a more integrated approach to regulation, research, and intellectual property protection across the continent. More recently, the African Network for Drugs and Diagnostics Innovation (ANDI), which aims to coordinate existing R&D in Africa towards addressing public health needs, is an example of how African stakeholders are taking leadership in managing and strengthening their own health innovation agenda [19].

This series in BioMed Central’s journal *BMC International Health and Human Rights* adds to these efforts by filling the gap in knowledge around Africa’s capacity for science-based health innovation, including biotechnology. By “science-based health innovation”, we mean technological innovation across a spectrum of sophistication, from vaccines, pharmaceuticals, and medical devices, to some plant medicines where attempts to scientifically standardize or characterize medicines have been made. We take a broad definition of innovation to refer not only to ‘new to the world’ technologies but also to the diffusion, adaptation and use of technologies [20]. We also use the OECD definition of biotechnology: “the application of science and technology to living organisms, as well as parts, products and models thereof, to alter living or non-living materials for the production of knowledge, goods and services” [21].

This series presents the results of extensive on-the-ground research in the form of four country case studies of health and biotechnology innovation, six studies of institutions within Africa involved in health product development, and one study of health venture funds in Africa. To the best of our knowledge it is the first extensive collection of empirical work on African science-based health innovation.

The articles are arranged with the country papers coming first to give overall context, followed by the institutional papers. The four country cases are Ghana, Rwanda, Tanzania and Uganda. These countries were chosen for a variety of reasons: their relatively high GDP among the larger sub-Saharan  African countries and, for the most part, stable governance situations; the presence of established health research institutes; and government policies supporting science for economic and social gains. The researchers also had invitations from government representatives to undertake studies.

The six case studies of institutions involved in health technology development are A to Z Textiles (Tanzania), Acorn Technologies (South Africa), Bioventures venture capital fund (South Africa), the Malagasy Institute of Applied Research (IMRA; Madagascar), the Kenyan Medical Research Institute (KEMRI; Kenya), and Niprisan’s development by Nigeria’s National Institute for Pharmaceutical Research and Development and Xechem (Nigeria). These case studies of institutions and products were chosen to illustrate relevant issues across regions and different types of institutions. They represent initiatives that have each achieved success in developing health solutions, albeit accompanied by important challenges and shortcomings. Together, the six institutional cases shed light on the dynamics and challenges at the level of individual institutions involved in African health innovation. Very often, these are shaped by the overall environment for innovation in the country; as such, the country papers provide context for the institutional papers, which in turn illustrate specific issues raised in the country papers. Each institutional paper also highlights one or more general policy questions facing those who would develop policy for, take part in, or invest in African-led health innovation.

South Africa is excluded from our country analysis because it is considerably more technologically advanced in health and biotechnology innovation than the rest of Sub-Saharan Africa, and because it has already been extensively reported upon elsewhere [22]. However, we include two institutional cases from South Africa (Acorn Technologies and Bioventures venture capital fund) as local pioneering examples of a life sciences incubator and fund respectively. Such incubators and funds are still nascent in the rest of sub-Saharan Africa, but are being actively explored, and we believe Acorn and Bioventures hold important lessons and cautionary notes for other sub-Saharan African countries.

We expect the collective results of this study to be of interest to a wide range of stakeholders including domestic governments; donor agencies; regional and international organizations seeking to promote science, technology and innovation in Africa; companies and researchers looking for partners; and those interested in investing in Africa.

## Methodology

Our conceptual framework for these studies is that of the ‘innovation system’, which holds that innovation is not a solitary or linear process, but rather involves learning and knowledge flow across a diverse network of stakeholders – firms, government departments, universities, non-governmental organizations, and end users [23,24]. As such, it focuses on interactions between groups, and the role of institutions and policy frameworks in enabling systemic performance. We recognize that the framework can only be taken as a guide when applied in the African context, and therefore have let the empirical data guide fieldwork and data analysis.

Our country and institutional case studies combine qualitative and quantitative data and use case study methodology. Data were collected through semi-structured face-to-face interviews with members of the public sector, R&D organizations, academia, government agencies, private sector enterprises, NGOs and funding agencies, supplemented with analyses of background documents and with observations. All quotes are from the interviews unless otherwise noted, and with permission. These studies were approved by the Office of Research Ethics of the University of Toronto.

## Summary of findings

Although differences exist across the countries in their environments for science-based health innovation—such as varying levels of scientific infrastructure, diverse policy instruments, and different levels of human resource development—in general, the core dynamics of innovation and challenges faced were similar. Likewise, the institutional cases, despite being drawn from a broad spectrum of countries and project types, suggest common themes. Below, we summarize key findings from our research.

### Despite challenges, components of health innovation exist

Our research finds a range of issues, generally common across all countries, that affect capacity for science-based health innovation. These include low levels of human resources with the skills to engage in research; low investment in scientific infrastructure and R&D (only Rwanda has an R&D investment over 1% GDP; Tanzania has recently pledged to raise its R&D investment to 1% of GDP as of 2009/2010); a research-focused public sector with weak ties to industry; little or no incentives for firms to innovate in the health area; and a lack of policy coherence so far on the use of science and technology (S&T) for development. However, we also found areas of institutional and research strength in each country, such as a wealth of experience in plant medicine, small entrepreneurial firms, regulatory expertise, government innovation funds, and scientists involved in well-regarded international research. Furthermore, as discussed in more detail in the country papers, country policies are gradually evolving towards incorporating innovation as well as S&T – Rwanda’s is focused on mobilizing its growing skilled population around key economic areas, and Ghana’s recently-published STI policy explicitly addresses innovation [25].

### Though limited, diverse activity in health innovation is occurring

All the countries we studied possess some examples of health innovation, generally in small, entrepreneurial firms. Activities include the production of essential medicines, such as anti-retrovirals, through technology transfer generally from other Southern firms; incremental innovations in making products better adapted to local conditions such as DanAdams’ pediatric anti-malarial formulation (Ghana) and Kampala Pharmaceutical Industries’s *Homapack* product for home-based management of children’s fever (Uganda). One firm – Ghana’s LaGray Pharmaceuticals – has the more advanced technological capabilities to produce the raw materials that go into drugs (Active Pharmaceutical Ingredients) and is the only such manufacturer in Sub-Saharan Africa outside of South Africa. Some capacity exists in diagnostics, and some in medical devices, such as A to Z (Tanzania), Africa’s largest bed-net manufacturer. Finally, we identify institutions and firms bringing scientific rigor to the commercialization of plant medicines. In general, strong entrepreneurial drive, a good understanding of local demand and, in most cases, a connection into international networks to access technology, funding or markets has been behind the success of these firms. However, local connections to other firms or to research institutions are weak, and most firms work in relative isolation.

### Despite the relatively large amount of health research, very little has resulted in new products or technologies to address local disease

All countries had one or more public sector research facilities or universities regarded as national leaders in health research. These were heavily supported by international funding, and networked into a range of international universities, donors and non-governmental organizations supporting health research. However most projects were at the basic or at best applied research level, and not concerned with the generation of local intellectual property or organizations to take new health solutions to scale. Where health research capacity is directed towards product development, it is usually through participation in clinical trials. As such, we found a range of ideas for, and sometimes even prototypes of, locally-relevant, health-focused technologies, which had stagnated within institutions which lacked the capacity to evaluate or further develop them. Examples include diagnostic tools such as Hepcell, a diagnostic kit for detecting human hepatitis B surface antigen (KEMRI, Kenya) and a rapid diagnostic test for schistosomiasis (Noguchi Institute, Ghana), as well as a wide variety of plant-based remedies at various stages of development. See Table 1 for a list of prospective technologies suitable for licensing and potential further development. Our case study of Kenya’s KEMRI demonstrates that an institution can be successful in doing research and building a network of international partnerships, but still struggle to turn its research into health solutions or technologies. Reasons for this are many, and include incentive structures within public research institutions, a skepticism towards commercial applications of knowledge, and the fact that health research can be driven by donor priorities and timelines rather than local ones. This is demonstrated by a lack of demand from end-users, particularly the local private sector, for the type of health research being conducted.

**Table 1:** Health technology stagnation in sub-Saharan Africa

	Technology	Description of technology (according to the scientist)*	Health area of application	Country / Institution	Status (according to the scientist)
**Traditional plant technologies**					

1	Artemisinin/lemon grass combination	A beverage that is used to treat malaria.	Malaria	Uganda- Natural Chemotherapeutics Research Laboratory	Ready for commercialization, but not yet commercialized. Undergoing clinical trials in northern Uganda
2	Nibima- Extract of traditional plant *Cryptolepis sanguinolenta*	Whole plant extract for the management of malaria	Malaria	Centre for Scientific Research into Plant Medicine, in Mampong, Ghana http://www.asnapp.org/	Needs validation through clinical trials
3	Neem tree	Extract from the Neem plant	Malaria	Kenya-ICIPE http://www.icipe.org	Scientist has no plans to commercialize. Needs validation through clinical trials.
4	Neem tree	Extract from the Neem plant	Malaria	Rwanda-IRST http://www.irst.ac.rw/	Scientist was unsure how to commercialize. Needs validation through clinical trials.
5	Whole plant extract	Whole plant extract	Malaria	University of Lagos http://www.unilag.edu.ng/index.php?page=home	Scientist was unsure how to commercialize. Needs validation though clinical trials.
6	Tanzed Plus	Whole plant extract for the management of HIV	HIV/AIDS management	Tanzania-Muhimbili University of Health and Applied Sciences http://www.muchs.ac.tz	Needs validation through clinical trials
7	Sunguprot	Extract of the root of *Tylosema Fassoglensis* mixed with soya beans-Nutritional supplement that acts as an immune booster.	HIV/AIDS management	Kenya-KIRDI http://www.kirdi.go.ke/	Needs validation through clinical trials
8	Morsella	A mixture of *Moringa oleifera* and *hibiscus sabdariffa*. Nutritional supplement that acts as an immune booster.	HIV/AIDS management	Tanzania –MUHAS *http://www.muchs.ac.tz/*	Needs validation through clinical trials
9	Plants extract	Treatment for opportunistic infections of HIV	Kaposi’s Sarcoma	Kenya-KEMRI www.kemri.org	Applied for patenting. Looking for commercial investor.
10	Whole plant extract	Treatment of Fibroids in women	Fibroids	Kenya- Moi University http://www.mu.ac.ke/	It is undergoing tests in humans in the form of pill formulations (Phase 1 clinical trials)
11	Plant extract	Has anti-sickling properties which are attributed to its ability to prolong or delay the time to polymerization of deoxy-Hb	Sickle cell anemia management	Nigeria-NIPRD http://www.niprd.org/	Orphan drug status by the US Food and Drug Administration (FDA) and the European Medicine Evaluation Agency (EMEA) in 2005. http://www.highbeam.com/doc/1G1-137075135.html Limited commercialization but failed to scale up because of business failure.
12	Anti-Hepatocyte	Plant derivative-A treatment of liver infections especially liver cirrhosis	Liver ailments	Tanzania-NIMR http://www.nimr.or.tz/	Scientist was unsure how to commercialize. Needs validation though clinical trials.
13	Aloe vera derivatives	Skin ointment	Skin ailments	Tanzania –MUHAS http://www.muchs.ac.tz/	Needs validation through clinical trials
14	Aloe vera derivatives	Skin ointment	Skin ailments	Rwanda-IRST http://www.irst.ac.rw/	Scientist was unsure how to commercialize. Needs validation through clinical trials.
15	Plant extract	Prevents the effects of radiation during x-ray's hence preventing cancers	Anti-radiation	Nigeria-University of Ibadan	Scientist was unsure how to commercialize. Needs validation through clinical trials.
16	Mondia Tonic	Root of *mondia whytei*	Anti-depressant	Kenya-ICIPE http://www.icipe.org	Partly commercialized. Needs validation through clinical trials to scale up commercialization.

**Diagnostics**					

17	Monoclonal antibody test for detection of malaria	A dipstick that uses antibody antigen reactions to detect presence of malaria parasites in the body by testing urine	Malaria	Ghana-University of Ghana http://www.ug.edu.gh/	Still being developed. Has received funds from the Gates foundation to explore the concept further.
18	Monoclonal antibody test for detection of Schistosomiasis	Rapid visually read monoclonal antibody (MoAb) based dipstick	Schistosomiasis	Ghana- Noguchi Memorial Institute for Medical Research http://www.noguchimedres.org/	Scientist has not attempted to commercialize. Ready for commercialization.
19	ELISA	A quick test for identifying MDR TB in sputum	Tuberculosis	Uganda-Makerere University http://mak.ac.ug/	No incentive to commercialize hence no product form developed.

**Medical equipment and associated health technologies**					

20	Medical waste incinerator	A fuel free medical waste incinerator. Suitable for destruction of plastics. Uses medical waste as fuel by generating very hot gases.	Medical waste	Uganda-Makerere University http://mak.ac.ug/	WHO approved. Commercialized but needs scaling up.
21	Female sanitary towels	Sanitary towels developed from lemon grass. Cost ¼ of conventional sanitary towels and suitable for school girls	Sanitary towels	Uganda-Makerere University http://mak.ac.ug/	The test was undergoing more evaluation.
22	Insect repellant	Use of human odors to repel malaria causing mosquitoes	Malaria	Kenya-ICIPE http://www.icipe.org	Patented-Ready for commercialization but formulation not developed.
23	Insect repellant	Plant extract	Malaria	Tanzania-NIMR http://www.nimr.or.tz	Needs validation through clinical trials.
24	TBCide	Disinfectant. Standardized chlorine based decontaminant used to destroy MDR TB.	Tuberculosis. resistant bacteria on surfaces bacteria from hospital surfaces.	Kenya-KEMRI www.kemri.org	Patented-Ready for commercial exploitation
25	Alternative methodology to extract and purify Artemisinin	To prepare derivatives or combine it with other anti-malarials to combat Artemisinin resistance	Malaria	Tanzania-NIMR http://www.nimr.or.tz	Needs extraction equipment and equipment for further validation

*Scientists interviewed were asked not to disclose any potentially proprietary information.  Reprinted from Simiyu, Ken *et al***.*** Science*; **330**: 1483-1484, 10 December 2010*.*

### All countries put strong emphasis on plant medicine as a local asset for innovation

Every country has institutions responsible for scientific research into plant medicines and cites plant medicine as a primary asset for African countries to enter ‘world first’ innovative activity in health product development. However, despite the enthusiasm for developing plant medicines to higher scientific standards, progress has been slow and the necessary coordination across groups absent. This signals the challenging nature of the field, where the communal aspect of knowledge ownership, variety of stakeholders involved, and lack of scientific infrastructure all play a part. A partial exception is Nigeria’s anti sickle cell drug Niprisan, whose development from a traditional medicine concoction was spearheaded by its National Institute for Pharmaceutical Research and Development (NIPRD) and commercial partner Xechem. Although its commercialization experienced difficulties, the case study highlights the importance of effective leadership and management, international partnerships, and government investment, and indicates where challenges might arise. Firms and institutions focused on commercializing plant medicines for local or regional markets only are more successful; the IMRA (Institut Malgache de Recherches Appliquées) institution in Madagascar, for example, illustrates a decades-old institution that has adapted and scaled up traditional remedies, though mostly for the local populace to date.

### Local, regional and global dynamics are affecting health innovation

National institutions, infrastructure and policies set the basic context for science-based health innovation. However, our case studies found that regional and global influences are at least as important in Africa. At a regional level, access to regional markets is a growing concern for firms and is seen by many as the ‘make or break’ factor in moving from imitative into innovative activity. Investing in regional drug-development infrastructure in order to share costs and centralize expertise was also cited by firms hoping to manufacture drugs to global standards. At a global level, South-South partnerships are playing a key role in technology transfer and capability-building, particularly in essential medicines [26]. Diaspora scientists and managers who are returning to their countries are often those pushing the boundaries of health innovation, and bringing with them global contacts and knowledge [27]. International donors strongly influence a range of areas pertinent to innovation: shaping research agendas, assisting with capacity building in STI, and often forming the major markets for products. Finally, global regulation – most prominently intellectual property – continues to shape national policy, with a global debate underway on which intellectual property regimes and incentives will best support research and innovation in areas such as neglected diseases [28,29]. Therefore, African governments must also be active at regional and global levels to ensure that local health needs can be met, including influencing donor policies to best generate locally-relevant and locally-owned knowledge.

### Institutions used innovative financing mechanisms and partnerships to their benefit

Our country case studies highlighted the many constraints to firms working in health innovation, and demonstrated how access to financing and to knowledge through partnerships were key elements to their success. This was also demonstrated in all six institutional cases. Means of financing covered a wide spectrum: A to Z combined co-investment from a Japanese multinational with its own family funding; Bioventures employed investment from the International Finance Corporation and other developmental institutions; the Niprisan project gained both private and public funding from Nigeria and the U.S., as well as indirect financial benefits from Orphan Drug legislation. Similarly, partnerships played a key role in each case: IMRA turned its low-resource setting into a strength by playing the role of an authority on local biodiversity to international researchers; KEMRI benefited from the Japanese institution JICA as well as the Wellcome Trust and many research partnerships; Acorn’s virtual model used linkages with external talent to incubate and support its investees. Implications are that financing and partnerships are key to successful development of African health solutions; that such financing and partnerships come in many forms, some far from obvious; and that supportive platforms that help enable financing and partnerships, and make such opportunities clearer to domestic and international actors, are a cross-cutting means of support that may benefit all parties. Moves are already being made in this direction, such as Uganda’s Window C and Presidential Funds which encourage public-private collaboration.

### Linkages between groups are sparse to date, but hold potential for building stronger health innovation systems

Taking a systemic approach to health innovation in Africa – i.e examining linkages between groups and seeking to understand the context affecting health innovation – reveals interesting results and rich areas for future enquiry. We found a range of institutions and policies that, on the surface, are relevant to science-based health innovation in Africa. However, often they have been modeled on foreign examples and have not found a natural place within the system, remaining isolated and without function. Relatedly, a range of reasons such as lack of trust exist for why stakeholders are not interacting, which need to be addressed if science-based health innovation is to develop. For example, intellectual property protection is unlikely to be utilized if firms do not trust the state to enforce it, and innovation policies are only valuable if there are means to mobilize stakeholders around their implementation. On the other hand, we see that important, organic linkages do exist – between African firms and other Southern firms, between firms and consumers, and between universities and local communities in terms of knowledge transfer in health – which indicate the beginnings of systemic interaction which can be built upon. Activities such as cluster-building in Tanzania, and business incubation in Uganda and South Africa, illustrate mechanisms that can bring together business support, mentorship, funding, and networking to help nascent companies develop their ideas and business acumen.

## Concluding remarks

The research presented in the series highlights compelling examples of African-led health innovation, including Tanzania’s A to Z, Nigeria’s Niprisan, LaGray’s local production of Active Pharmaceutical Ingredients, and several investments in scientifically upgrading plant medicine. For the most part, these are not ‘world-first’ innovations, but rather Africa-firsts, involving the adoption and adaptation of technologies to become more affordable, convenient and suitable to African conditions. However some examples, such as the sickle-cell drug Niprisan, are world firsts.

All of the examples highlight pioneering attempts to build technological capacity, create economic opportunities, and retain talent on a continent significantly affected by brain drain. The examples are largely “one-off’s”, pursued by highly motivated individuals with generally weak support networks. They point to the practical challenges for innovators on the ground, and suggest potentially helpful policies, funding streams, and other support systems.

Most importantly, perhaps, they illustrate the diversity of types of health innovation that are occurring in Africa – each of which utilize different competencies, resources, and strategies, depending on the technology, the market, the investment, the source of knowledge, and other factors. As such, clarifying the best strategies to support health innovation will be critical to African countries, both individually and working across regions. Some of these strategies in the context of investment and market opportunities for African health innovation have been set out elsewhere [30]. An area for future research is to develop an interactive “opportunity guide” with which countries and institutions can explore appropriate health innovation opportunities, according to their resources, goals, and competencies – building on work already underway for pharmaceutical innovation in Africa [31].

One compelling argument for local innovation relates to the fact that much of the prevalent disease burden could be mitigated through locally relevant and affordable diagnostics, drugs, and devices, and more generally through better public health infrastructure and complementary health delivery systems – all of which are culturally specific. The value of harnessing local insights, then, goes beyond keeping profits and capabilities in Africa – it also has direct potential to customize novel prevention and treatment methods, so that diseases are addressed at scale more effectively. Indeed, much of the health innovation chronicled in these papers has come about through intimate knowledge of local markets, generation of local health delivery systems, and similar tacit knowledge that comes from “learning by doing”; such “innovation capital” may not be captured well by traditional metrics such as patents and researchers per capita. All of these considerations suggest a uniquely African approach toward health innovation that may emerge.

A related challenge is moving African health innovation from a collection of one-off success stories to a set of sustainable innovation systems: KEMRI, Niprisan, and Acorn are examples of cases illustrating the difficulties involved. Greater understanding and capacity is needed to understand financial and organization models sustainable in the long term, and incentives for institutions and policy-makers to adapt and refine these models. Flexibility to design such models and create enabling policies will also be needed, and donors should be cognizant of this. The Ghanaian attempt to address stimulate microbusinesses around health needs, which floundered due to lack of resources and pressures from competing priorities, is a case in point. Funding is a core requirement for sustainability, and it is worth noting that many of the countries discussed do have precedents in the form of domestic and international investment into low-risk health delivery ventures like hospitals. There may be potential to channel some of this funding to lower-risk R&D, which could complement venture funding and international health R&D dollars for earlier-stage or more risky R&D, e.g. as part of product development partnerships.

An important element for success will be devising and supporting strategies which energize diverse parts of the innovation system together around specific goals and realistic aims. An example would be a strategy to bring together the three most important elements which are disconnected so far – science, business and capital. We have proposed the idea of an innovation platform to do this, combining physical and virtual elements [32,33]. A network of such platforms could provide a single point of entry for developmental and private investors interested in supporting innovation in Africa.

For African nations, health innovation represents an opportunity to increase domestic capacity to solve health challenges; for international funders, it is an opportunity to move beyond foreign aid and dependency. The shared goal is creating self-sustaining innovation that has both health and development impacts. This is a long-term strategy, and we do not underestimate the difficulties. At the same time, this series shows the potential of African-led innovation, and indicates how it might balance realism against opportunity. There is scope to learn lessons more systematically from cases like those we discuss; to link entrepreneurs, scientists, funders, and policy-makers into a network to share opportunities and challenges; and ultimately to better support and stimulate African-led health innovation.

At its core, innovation is about taking knowledge and making it work for a society. African nations may not be able to reinvent themselves as knowledge economies overnight. They can, however, take realistic steps in this direction, while benefiting in both health and economic terms. External funders and partners, by supporting African-led innovation, can help to make the influx of funding more fair, respectful, and sustainable, leading to greater capacity and autonomy in recipient countries.

## Competing interests

The authors declare that they have no competing interests.
